# Supplementary material for: Awareness of management of pulmonary multidrug-resistant tuberculosis (MDR-TB) among private practitioners in suburban areas of Pune city, India: Input for developing an educational tool
Source: PLOS Glob Public Health. 2025 Sep 17;5(9):e0004617. doi: 10.1371/journal.pgph.0004617 (PMC12443276; doi:10.1371/journal.pgph.0004617)
Supplement: Attachment — Submitted filename: Response to reviewers.docx [file pgph.0004617.s002.docx]

**Journal Requirements:**

**1. Please include a complete copy of PLOS’ questionnaire on inclusivity in global research in your revised manuscript. Our policy for research in this area aims to improve transparency in the reporting of research performed outside of researchers’ own country or community. The policy applies to researchers who have travelled to a different country to conduct research, research with Indigenous populations or their lands, and research on cultural artefacts. The questionnaire can also be requested at the journal’s discretion for any other submissions, even if these conditions are not met. Please find more information on the policy and a link to download a blank copy of the questionnaire here:** [**https://journals.plos.org/globalpublichealth/s/best-practices-in-research-reporting**](https://journals.plos.org/globalpublichealth/s/best-practices-in-research-reporting)**. Please upload a completed version of your questionnaire as Supporting Information when you resubmit your manuscript.**

Response: We have included a complete copy of PLOS’ questionnaire on inclusivity in global research in your revised manuscript.

**2. Please amend your detailed Financial Disclosure statement. This is published with the article. It must therefore be completed in full sentences and contain the exact wording you wish to be published.**

**i. Please clarify all sources of funding (financial or material support) for your study. List the grants (with grant number) or organizations (with url) that supported your study, including funding received from your institution. 
ii. State the initials, alongside each funding source, of each author to receive each grant.
iii. State what role the funders took in the study. If the funders had no role in your study, please state: “The funders had no role in study design, data collection and analysis, decision to publish, or preparation of the manuscript.”
iv. If any authors received a salary from any of your funders, please state which authors and which funders.**

**Response:** Financial Disclosure statement has been amended and included at the end of the discussion section after the acknowledgements.

**3. Please make sure the funding information on the submission form matches your financial disclosure statement. Please indicate by return the full and correct funding information for your study and confirm the order in which funding contributions should appear. Please be sure to indicate whether the funders played any role in the study design, data collection and analysis, decision to publish, or preparation of the manuscript.**

**Response:** Financial Disclosure statement has been amended and included at the end of the discussion section after the acknowledgements.

**4. Please insert an Ethics Statement at the beginning of your Methods section, under a subheading 'Ethics Statement'. It must include:**

**1) The name(s) of the Institutional Review Board(s) or Ethics Committee(s)**

**2) The approval number(s), or a statement that approval was granted by the named board(s)**

**3) (for human participants/donors) - A statement that formal consent was obtained (must state whether verbal/written) OR the reason consent was not obtained (e.g. anonymity). NOTE: If child participants, the statement must declare that formal consent was obtained from the parent/guardian.**

Response: Ethics Statement has been included at the beginning of Methods section.

**5. In the online submission form, you indicated that “Data are available with the first author. Required data are presented in the form of tables.”.**

**All PLOS journals now require all data underlying the findings described in their manuscript to be freely available to other researchers, either 
1. In a public repository, 
2. Within the manuscript itself, or 
3. Uploaded as supplementary information.**

Response: The full data file has been uploaded as Supplementary Information.

**Response to reviewers (Please refer to the page numbers in the revised manuscript with tracked changes)**

**Reviewer #1: Among the key words include India**

Response: Thank you. We included “India” as the key word (Page 3, Line 56).
 **Line 60: The TB case notifications increased by 9% (from 7.5 million in 2022 to 8.2 million in 2023) making TB the again the lead infectious killer surpassing COVID-19. Consider rewriting this sentence. It is not coherent.**
Response: Thank you.

The text is revised as: (Page 4 Lines 64-70)

While TB notification can serve as a proxy indicator for the incidence in countries with robust reporting systems as acknowledged by the WHO Global TB Report, it is not always the case particularly in high burden countries because underreporting from the private sector remains a major issue. The Global report further indicates that during COVID-19 pandemic, TB was sidelined- a reflection of which may be seen as 9% increase (from 7.5 million in 2022 to 8.2 million in 2023) in TB case notifications.

**Line 78: WGS resistance diagnosis as it cuts costs and turn-around time. I would think that this may not be entirely true. Please rewrite this sentence.**

Thank you. We revised the sentence as follows (Page 5; Lines 87-91)

“In several well-resourced settings, whole genome sequencing (WGS) is now routinely used for drug resistance diagnosis as it reduces the turn-around time and excludes the need for conventional stepwise drug susceptibility testing (DST), which delays an accurate diagnosis.”

**Line 139: Our team approached a total 123 PPs, of which we could enrol 100 in this study. What is the estimated total number of PP in Pune city, India so we can know the proportion represented by 100 PPs. I would have liked to know the number of suspected TB patients they see on average daily or monthly.**
Response: Thank you. We have now included this information and stated in the limitation of the study as well.

(Page 7: Lines 136-142)

In PCMC area, there are approximately 400 allopathic practitioners and 500 practitioners who practice alternative systems of medicine (e.g. Ayurveda, Homeopathy, Unani etc.) [20]. Since the aim of this assessment was to help design an educational tool and resources to support improved TB care, we predominantly included PPs practicing allopathic medicine who often diagnose and/treat TB and MDR-TB cases. We applied non-probability (purposive and snowball) sampling method for selecting 100 PPs that included general practitioners and chest physicians.

Page 18: Lines: 355-359

While our study offers insights to awareness of PPs regarding pulmonary TB/MDR-TB management, it has several limitations. First, we primarily sampled approximately 21% (85 of 400) of allopathic PPs from the study area who actively manage and/or notify TB cases to the NTEP. However, the number of PPs from alternative systems in the study area as reported in a previous study is large [20].

The suspected number of TB patients PPs see/notify was not the part of inquiry of this study. However, our earlier study from the same setting published in the Indian Journal of TB reports that 92% of PPs notify <2 patients in a month, 6% PPs notify 3-10 patients and 2% report >10 patients per month.

(Ref: Sahasrabudhe T, Barthwal M, Sawant T, Ambike S, Jagtap J, Hande S, Atre S. Tuberculosis notification: An inquiry among private practitioners in Pimpri-Chinchwad municipal corporation area of Maharashtra, India. Indian Journal of Tuberculosis. 2022 Jan 1;69(1):73-8.)

**Line 178: further, the research team found that some PPs were erroneously referring ‘Xpert assay’ as ‘WGS’. What percentage of PPs was this?**

Response: Lines 209-214: We than the reviewer for this comment.

However, when asked about the turnaround time required for getting the WGS test results, 6 reported >14 days, whereas 7 reported between 7-14 days. When inquired further, the research team found that remaining 3 PPs erroneously referred ‘Xpert assay’ as ‘WGS’ as they reported receipt of report within <6 days (which is generally Xpert assay report). Thus, 13 of 37 PPs reported that they refer patients for the WGS.

**Discussion

I am interested in comparing the turnaround time for TB diagnosis between private and government facilities. Please if you can provide details on this.**

Response: We thank the reviewer for raising this point. However, this information was not collected as a part of this study. Nevertheless, our earlier study published in the American Journal of Respiratory and Critical Care Medicine in 2022 has reported some results. With this reference, we added text in the discussion.

Page 15-16: (Lines 296-305]:

Furthermore, the lack of awareness about Xpert assay can result in delayed diagnosis of TB and MDR-TB which eventually can lead to transmission of resistant TB strains in the communities. Our previous study supports this assertion. It reported the median diagnostic delay (onset of symptoms to diagnosis) for patients with MDR-TB as 90 days (IQR 60-180) and 68% (87 of 128) of them reported private sector as the first point. Similarly, even among non MDR patients the median diagnostic delay was 60 days (IQR 30-90) and for 100 of 139 private sector was the first point of help seeking [17]. In case of government sector/the NTEP, the diagnosis of TB and RR/MDR-TB generally happens within 3 days, and things have improved especially after the rollout of Xpert assay.

**Also comment on the standard of diagnosis for MDR-TB in India**

Following lines are added in the discussion page 14: lines 276-277

The standard of diagnosis for MDR-TB in involves clinical suspicion and rapid molecular testing for rifampicin resistance using Xpert assay.

**Comment about the cost of treating MDR TB in PP versus government.**

Thank you for the comment. We added the text and reference of a study

Page 15: Lines 289-291

A study by Mullerpattan et al. in Mumbai reported that treatment cost for pulmonary MDR-TB in the private sector was exorbitant with an average $5723 [25].

**Reviewer #2: The manuscript is well-written and presents an important issue regarding the role of private practitioners in TB care in India. The research topic is highly relevant to public health, especially considering the significant burden of TB and MDR-TB in the country.**

We thank the reviewer for the comment.

**However, I have two main concerns that need to be addressed:

First, the sample size of 100 private practitioners seems relatively small, which may limit the generalizability of the findings. In the methodology section, you should provide a more detailed description of the sampling process. How were the participants selected? What measures were taken to ensure the representativeness of the sample? A clearer explanation of the sampling strategy would strengthen the study's credibility.**

Response: Thank you for the comment. The first reviewer raised same comment. Hence the response is also same as above.

(Page 7: Lines 136-142)

In PCMC area, there are approximately 400 allopathic practitioners and 500 practitioners who practice alternative systems of medicine (e.g. Ayurveda, Homeopathy, Unani etc.) [20]. Since the aim of this assessment was to help design an educational tool and resources to support improved TB care, we predominantly included PPs practicing allopathic medicine who often diagnose and/treat TB and MDR-TB cases. We applied non-probability (purposive and snowball) sampling method for selecting 100 PPs that included general practitioners and chest physicians.

Page 18: Lines: 355-359

While our study offers insights to awareness of PPs regarding pulmonary TB/MDR-TB management, it has several limitations. First, we primarily sampled approximately 21% (85 of 400) of allopathic PPs from the study area who actively manage and/or notify TB cases to the NTEP. However, the number of PPs from alternative systems in the study area as reported in a previous study is large [20].

**Second, there is a lack of discussion on study limitations: The discussion section does not adequately acknowledge the study's limitations. It is important to acknowledge potential limitations such as the small sample size, potential selection bias, and any other factors that may have influenced the results. Discussing these limitations will provide a more balanced interpretation of the findings and demonstrate a deeper understanding of the research's strengths and weaknesses.**

Response: We thank the reviewer for this point. We added a section on the limitations as suggested.

**Limitations of the study** (Page 18: Lines 355-369)

While our study offers insights to awareness of PPs regarding pulmonary TB/MDR-TB management, it has several limitations. First, we primarily sampled approximately 21% (85 of 400) of allopathic PPs from the study area who actively manage and/or notify TB cases to the NTEP. However, the number of PPs from alternative systems in the study area as reported in a previous study is large [20]. Due to limited funding and short duration of the study, we had to adapt non-probability sampling method. Thus, our study is prone to selection bias to some extent. Second, there are numerous PPs belonged to alternative systems such as Ayurveda and Homeopathy but manage TB cases through allopathic practice. Given the limited resources, we included only a few known PPs from them. Thus, we could get their limited insights. Third, we did the study among PPs in urban areas. Thus, the generalisability of the findings may be limited. Nevertheless, studying awareness among PPs and their practices related to TB/MDR-TB care in rural areas where the access to information and technology is limited would be important. We strongly believe that the identified common gaps and suggested measures will help in contributing to the development of an educational tool that will have wider applicability.
